# Can Posterior Pericardial Incision Truly Improve Postoperative Complications After Cardiac Surgery? A Systematic Review and Meta-Analysis

**DOI:** 10.21470/1678-9741-2022-0350

**Published:** 2023-07-14

**Authors:** Zhe-an Shen, Yingze Hou, Limei Yu, Xiaofang Wang, Aiqiang Dong, Minjian Kong, Heng Shi

**Affiliations:** 1 Department of Cardiovascular Surgery, The Second Affiliated Hospital, Zhejiang University School of Medicine, Hangzhou, People’s Republic of China; 2 Department of Biomedical Research, Research and Innovation Center, Xinjiang Institute of Technology, Xinjiang, People’s Republic of China; 3 Sanquan College of Xinxiang Medical University, Xinxiang, People’s Republic of China; 4 Hangzhou Traditional Chinese Medicine Hospital Affiliated to Zhejiang Chinese Medical University, Hangzhou, People’s Republic of China.

**Keywords:** Pericardiotomy, Atrial Fibrillation, Cardiac Surgery, Postoperative Care, Meta-analysis

## Abstract

**Introduction:**

Postoperative atrial fibrillation (POAF) and pericardial effusion are important factors affecting prognosis after cardiac surgery. Recently, it has been reported that posterior pericardiotomy (PP) can effectively prevent the occurrence of POAF and pericardial effusion. To validate these conclusions and guide clinical practice, we conducted a systematic review with meta-analysis.

**Methods:**

We searched multiple databases for manuscripts published before July 2022 on the use of PP to prevent POAF and pericardial effusion and included only randomized controlled trials. The main outcome was atrial fibrillation after coronary artery bypass grafting, and secondary outcomes were included.

**Results:**

This meta-analysis included 14 randomized controlled trials with a total of 2275 patients. Meta-analysis showed that the incidence of POAF after cardiac surgery in the PP group was significantly lower than that in the control group (risk ratio=0.48; 95% confidence interval=0.33~0.69; P<0.00001). PP effectively reduced postoperative pericardial effusion (risk ratio=0.34, 95% confidence interval=0.21-0.55; P<0.00001).

**Conclusion:**

PP has shown good results in preventing POAF, pericardial effusion, and other complications, which indicates that PP is a safe and effective surgical method, but attention still needs to be paid to the potential risk of coagulation dysfunction caused by PP.

## INTRODUCTION

**Table:** 

Abbreviations, Acronyms & Symbols				
BO	= Before operation		LV	= Left ventricular
CABG	= Coronary artery bypass grafting		M-H	= Mantel-Haenszel
CHA₂DS₂-VASc	= Congestive heart failure, hypertension, age ≥ 75 years (doubled), diabetes, stroke (doubled), vascular disease, age 65 to 74 years, and sex category (female)		MVR POAF PP	= Mechanical valve replacement = Postoperative atrial fibrillation = Posterior pericardiotomy
CI CR	= Confidence interval = Coronary revascularization		PRISMA	= Preferred Reporting Items for Systematic Reviews and Meta-analyses
EF	= Ejection fraction		RCT	= Randomized controlled trial
FE	= Fixed effect		RE	= Random effect
IABP	= Intra-aortic balloon pump		RR	= Risk ratio
ICU	= Intensive care unit		SE	= Standard error

Recently, coronary heart disease has become a major cause of high morbidity and mortality worldwide^[1-3]^. Surgery is the only way to treat coronary heart disease when conservative treatment is ineffective, and the most common surgical procedure is coronary artery bypass grafting (CABG). Other cardiac surgeries, such as valve replacement, valvuloplasty, and atrial septal defect repair, are also widely carried out in hospitals around the world. Postoperative atrial fibrillation (POAF) is one of the most common complications after cardiac surgery, with an incidence of 20-40%^[2]^. POAF increases the possibility of heart failure and stroke and is an important factor affecting postoperative mortality^[4-7]^. Therefore, it is urgent to find a method to mitigate POAF. However, since the physiological mechanism of atrial fibrillation after cardiac surgery is not clear, generally only symptomatic and supportive treatment, such as the use of amiodarone and other drugs, is provided in clinical practice. However, the application of drugs is only a treatment measure, and mitigating the occurrence of POAF is still a major problem. Therefore, in 1995, Mulay et al.^[8]^ invented posterior pericardiotomy (PP), a simple surgical procedure to drain pericardial effusion into the pleural cavity through a posterior pericardial incision. Its core mechanism is to reduce POAF by draining pericardial effusion. To date, many reports have noted that PP can reduce the incidence of POAF and the presence of postoperative pericardial effusion. However, several of these findings are contradictory. For example, Kongmalai et al.^[9]^ reported that PP cannot reduce the incidence of POAF but may aggravate infection, increase drainage, and affect the prognosis time. Previously, a meta-analysis was conducted to evaluate posterior pericardial resection, but their assessment did not take into account the impact of pericardial effusion, and the literature was not fully included^[10]^. In addition, previous meta-analyses have high heterogeneity^[10]^. Also, several new high-quality randomized controlled trials (RCTs) have provided new data. Therefore, we conducted a meta-analysis of RCTs to systematically evaluate the improvement impact and effectiveness of PP on POAF and pericardial effusion after cardiac surgery and to provide deeper and more evidence-based guidance for clinical practice.

## METHODS

This systematic review and meta-analysis is based on the Preferred Reporting Items for Systematic Reviews and Meta-analyses (PRISMA)^[11]^. The work has been reported in line with PRISMA and Assessing the Methodological Quality of Systematic Reviews (or AMSTAR) Guidelines. The protocol for this systematic review was registered on PROSPERO (CRD42022350589).

### Search Strategy

The search strategy used the Population, Intervention, Comparison, Results and Research Design (or PICOS) criteria recommended in the Cochrane Handbook for Systematic Reviews^[12]^. We searched the PubMed®, Web of Science™, Embase, Cochrane Library, and China National Knowledge Infrastructure (or CNKI) databases, and the retrieval date was until July 2022. The search terms included “posterior pericardiotomy”, “CABG”, “coronary artery bypass grafting”, “PP”, “retropericardial incision”, “heart surgery”, “pericardial effusion”, “cardiac tamponade”, “atrial fibrillation”, “postoperative atrial fibrillation”, and “cardiac surgery”.

To search as many documents as possible and improve the quality of the retrieval, we did not set language restrictions. Also, to ensure the high quality of the retrieval, we chose to use artificial secondary screening. Two reviewers (ZA Shen and Y Hou) independently conducted a secondary screening, and the articles passed by the screening were formally included. If there was ambiguity between the two reviews, study inclusion was determined by the third reviewer (H Shi).

### Inclusion Criteria

We used the following inclusion criteria: (1) the research type was RCT; (2) adult patients (≥ 18 years) undergoing cardiac surgery; (3) there was a control arm (PP was performed or not performed); (4) clear indications for cardiac surgery; and (5) randomly assigned experimental group and control group.

### Exclusion Criteria

We also had strict exclusion criteria for data reliability: (1) clinical trials without ethical approval; (2) animal and *in vitro* experiments; (3) multiple organ failure in preoperative patients; (4) patients who underwent radiofrequency ablation; and (5) research with potential conflicts of interest.

### Data Extraction and Outcome Measures

The data extraction process was independently completed by two authors (Y Hou and Z Shen). The extraction contents included the first author’s name, publication year, experimental design, number of patients in the experimental group and the control group, baseline data of patients, treatment process, number of POAF patients, number of pericardial effusion patients, mortality rate, length of hospital stay, number of patients using an intra-aortic balloon pump (IABP), number of arrhythmia patients, and number of other complications.

The main outcome indicator was the occurrence of POAF, and the secondary outcome indicators included pericardial effusion, length of stay in the intensive care unit (ICU), occurrence of arrhythmia, use of IABP, length of stay, positive muscle support demand, and pleural effusion.

### Bias Risk Assessment

We used the Cochrane Risk Bias Evaluation Tool to evaluate the risk bias of the RCTs included. The evaluation points were as follows: random sequence generation (selection bias), allocation concealment (selection bias), blinding of participants and personnel (performance bias), blinding of outcome assessment (detection bias), incomplete outcome data (attrition bias), selective reporting (reporting bias), and other biases not mentioned above. The authors carefully assessed the risks of various types of bias and choose one of the three options. The assessment rules were as follows: high risk (the authors believe that the risk may or will affect the accuracy of subsequent data analysis), unclear risk (the authors were unable to objectively or correctly assess the risk of the bias for various reasons or the risk level of the bias was between high and low risk), and low risk (the authors believe that this bias does not affect the accuracy of subsequent data processing or is unlikely to affect it).

### Statistical Analysis

We used Revman 5.3 software (Cochrane Collaboration) and Stata 16.0 (Stata Corp LP) for meta-analysis. Risk ratio (RR) and 95% confidence interval (CI) were used as the comprehensive measurement standard of binary data. The range of the heterogeneity index (I2) was set to 0-100%. When I2 > 50, statistical heterogeneity was identified. When I2 < 50, we used the fixed effect (FE) model; otherwise, we used a random effect (RE) model to reduce unreliable outcomes due to high heterogeneity. When I2 > 50, we used sensitivity analysis or subgroup analysis to eliminate or explain potential strong heterogeneity. A two-tailed test level < 0.05 was considered statistically significant (*P*<0.05).

## RESULTS

### Study Selection and Characteristics

**Fig. 1:**
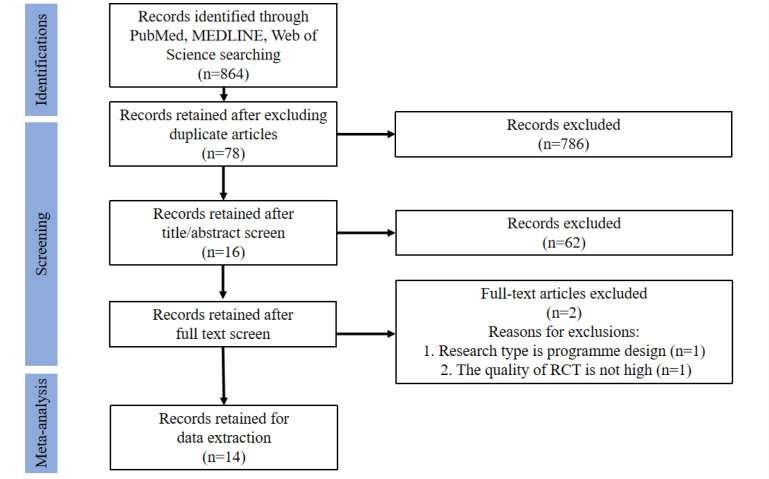
Flow chart of the search and selection process. RCT=randomized controlled trial.

We searched PubMed®, Web of Science™, and Embase databases. We found a total of 864 results, leaving 78 records after removing duplicates. Sixty-two records were excluded after title/summary screening. After evaluating 16 full texts, we excluded two because 1) the research type was program design and 2) the quality of the RCTs was not high, manifested in the absence of strict grouping, and the risk of classification bias in the control group and the experimental group was high. Figure 1 shows our retrieval strategy and results.

Finally, we identified 14 studies that met our inclusion criteria. These 14 studies were published between 1997 and 2021, with a total sample size of 2775. Table 1 shows the baseline data and characteristics of these 14 studies^[13-26]^. Ten RCTs evaluated the effect of PP on patients after CABG^[13-15,17,18,20,21,23,25,26]^. All studies except one included > 100 patients^[15]^.

**Table 1:** Baseline data of randomized controlled trials included in the meta-analysis.

Study	Study design	Surgery type	Number of patients (PP/control)	Age (years)		Sex (M/F)		Normal LV function (EF > 50%, BO)	
				PP	Control	PP	Control	PP	Control
Arbatli, 2002^[24]^	RCT	CR	113 (54/59)	62.3±8.2	60.1±9	45/9	44/15	21	28
Asimakopoulos, 1997^[26]^	RCT	CABG	100 (50/50)	61±9	61±2	None^*^	None^*^	38	32
Bakhshandeh, 2009^[22]^	RCT	CABG/MVR	410 (205/205)	67.3±8.2	68.2±9	78/127	86/119	46	27
Cakalagaoglu, 2012^[25]^	RCT	CABG	100 (50/50)	63.2±7.67	58.8±12.7	40/10	43/7	44	42
Ekim, 2006^[18]^	RCT	CABG	100 (50/50)	59.1±8.9	60.1±3.2	17/33	18/32	19	21
Erdil, 2005^[19]^	RCT	MVR	100 (50/50)	40.9±13.9	43.2±15.4	27/23	24/16	None^*^	None^*^
Farsak, 2002^[20]^	RCT	CABG	150 (75/75)	64.2±8.9	62.8±5.4	27/75	24/75	None^*^	None^*^
Fawzy, 2015^[13]^	RCT	CABG	200 (100/100)	54.3±8.6	56±9.7	64/36	68/32	87	82
Gaudino, 2021^[14]^	RCT	CABG	420 (212/208)	61±8	62±8	50/162	52/156	212	208
Haddadzadeh, 2015^[23]^	RCT	CABG	207 (105/102)	61.07±10.4	61.4±11.6	72/33	70/32	79	51
Kaya, 2014^[16]^	RCT	CABG	210 (107/103)	58.39±9.24	57.46±9.1	80/23	84/23	None^*^	None^*^
Kaygin, 2011^[21]^	RCT	CABG	425 (213/212)	58.8±11.3	59.0±11.3	107/106	105/107	98	105
Kongmalai, 2014^[15]^	RCT	CABG	20 (10/10)	64.9±13.11	59.2±4.69	5-mai.	5-mai.	8	5
Kuralay, 1999^[17]^	RCT	CABG	200 (100/100)	57±12	61±8	77/23	73/27	57	65

BO=before operation; CABG=coronary artery bypass grafting; CR=coronary revascularization; EF=ejection fraction; LV=left ventricular; MVR=mechanical valve replacement; PP=posterior pericardiotomy; RCT=randomized controlled trial

* Indeterminate

### Quality Assessment

**Fig. 2:**
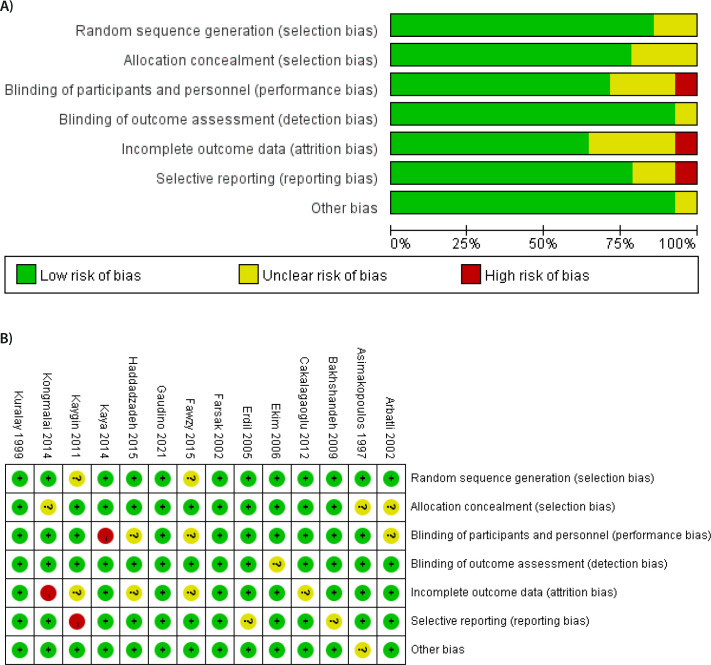
Bias risk assessment figure. A) Percentage diagram of each bias risk evaluation index. B) Bias risk assessment diagram of the included literature.

After risk bias assessment, three of the included studies^[14,17,20]^ were of high quality, and all the bias risks were assessed as “low risk”. One study^[16]^ did not clearly specify the blinding method used. Because relevant information was not provided, we believe that the risk of potential bias caused by the blinding method was high. In the study by Kongmalai et al.^[15]^, the results were partly unclear, and the number of outcome indicators was too low. One study^[21]^ was assessed as having high-risk reporting bias because it did not provide details of any adverse outcomes. Specific bias evaluation results are shown in Figure 2. The methodological evaluation of the included RCTs is shown in Table 2.

**Table 2:** Methodological evaluation of included randomized controlled trials.

Study	Randomization	Concealment	Blinding	Follow-up	Quality of evidence
Arbatli, 2002^[24]^	Yes	Yes, allocations were masked	Yes, single-blinded (investigators)	7 months	⨁⨁⨁○/Moderate
Asimakopoulos, 1997^[26]^	Yes	Yes, allocations were masked	Yes, single-blinded (investigators)	Not described	⨁⨁○○/Moderate
Bakhshandeh, 2009^[22]^	Yes	Yes, allocations were masked	Yes, single-blinded (investigators)	13 months	⨁⨁⨁○/Moderate
Cakalagaoglu, 2012^[25]^	Yes	Yes, allocations were masked	Yes, single-blinded (investigators)	10 months	⨁⨁⨁○/Moderate
Ekim, 2006^[18]^	Yes	Yes, allocations were masked	Yes, single-blinded (investigators)	22 months	⨁⨁⨁⨁/High
Erdil, 2005^[19]^	Yes, random number hiding method	Yes, allocations were masked	Yes, single-blinded (investigators)	21 months	⨁⨁⨁⨁/High
Farsak, 2002^[20]^	Yes, random number hiding method	Yes, allocations were masked	Yes, single-blinded (investigators)	18 months	⨁⨁⨁⨁/High
Fawzy, 2015^[13]^	Yes, random number hiding method	Yes, allocations were masked	Yes, single-blinded (investigators)	2 years	⨁⨁⨁⨁/High
Gaudino, 2021^[14]^	Yes, use CHA₂DS₂-VASc score	Yes, allocations were masked	Yes, double-blinded (subjects and investigators)	30 days	⨁⨁⨁⨁/High
Haddadzadeh, 2015^[23]^	Yes	Yes, allocations were masked	Not described	Not described	⨁⨁○○/Moderate
Kaya, 2014^[16]^	Yes, random number hiding method	Yes, allocations were masked	Not described	16 months	⨁⨁⨁○/Moderate
Kaygin, 2011^[21]^	Yes	Yes, allocations were masked	Yes, single-blinded (investigators)	18 months	⨁⨁⨁⨁/High
Kongmalai, 2014^[15]^	Yes	Yes, allocations were masked	Yes, double-blinded (subjects and investigators)	4 months	⨁⨁⨁⨁/High
Kuralay, 1999^[17]^	Not described	Yes, allocations were masked	Not described	1 year	⨁⨁○○/Moderate

CHA₂DS₂-VASc=congestive heart failure, hypertension, age ≥ 75 years (doubled), diabetes, stroke (doubled), vascular disease, age 65 to 74 years, and sex category (female) Methodological evaluation score/grade: ⨁○○○/Low, ⨁⨁○○/Moderate, ⨁⨁⨁○/Moderate, ⨁⨁⨁⨁/High. The higher the score, the more credible the study is.

### Primary Outcome: POAF

**Fig. 3:**
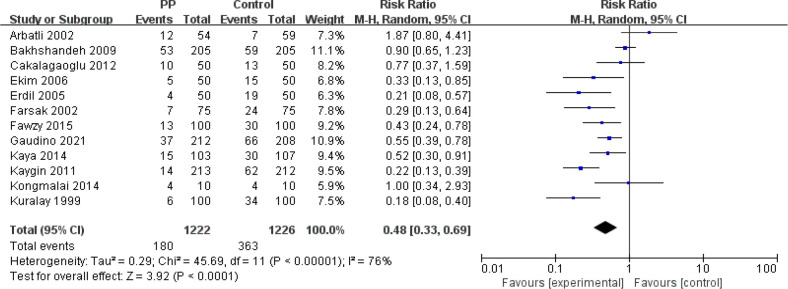
Forest plot showing the relationship between posterior pericardiotomy (PP) and postoperative atrial fibrillation. CI=confidence interval; M-H=Mantel-Haenszel.

RE was used for POAF, and 12 of the 14 RCTs^[13-22,24,25]^ we included reported this outcome. A total of 2448 participants (1222 in the PP group and 1226 in the control group) were included in this analysis. The incidence of POAF in the PP group was 14.7% and that in the control group was 29.6%. PP during cardiac surgery to reduce the incidence of POAF was effective (RR=0.48; 95% CI=0.33~0.69; *P*<0.00001). And strong heterogeneity was found among the studies (I2=76%, heterogeneity *P*<0.0001) (Figure 3).

Because of the high heterogeneity of this analysis, we conducted a sensitivity analysis. After replacing the RE model with the FE model, the heterogeneity did not change, and the *P*-value of the analysis did not change. We analyzed the sensitivity of different surgical procedures in this analysis. We found that there was no significant difference in heterogeneity between CABG and other surgical procedures (I^2^=76%, heterogeneity *P*<0.0001 *vs.* I^2^=62%, heterogeneity *P*=0.01).

### Subgroup analysis: Pericardial effusion

**Fig. 4:**
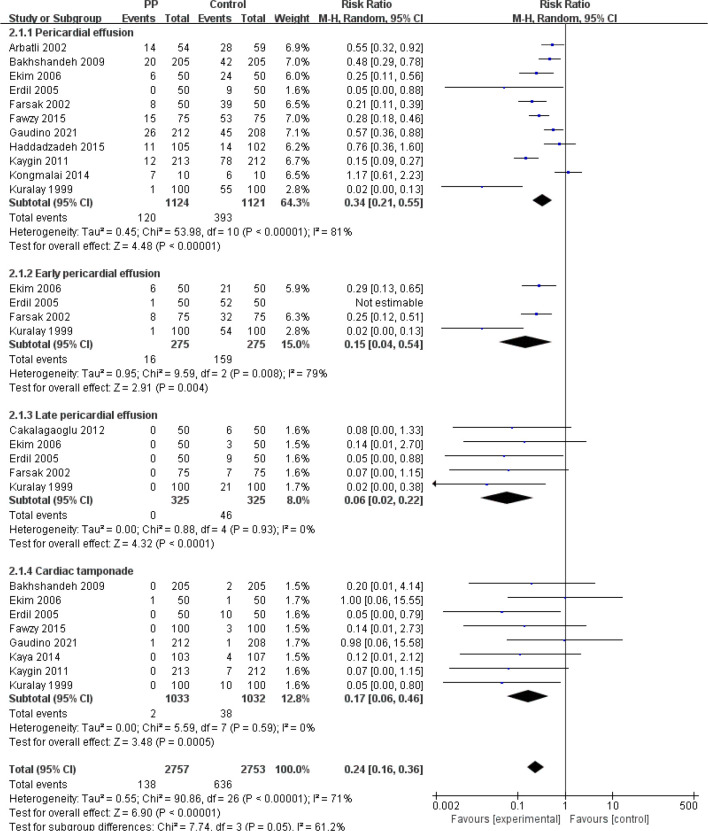
Subgroup analysis showing the effect of posterior pericardiotomy (PP) on pericardial effusion in different stages after cardiac surgery. CI=confidence interval; M-H=Mantel-Haenszel.

Eleven studies reported pericardial effusion. When evaluating the effect of PP on pericardial effusion, we found that the heterogeneity was too large, so we used subgroup analysis to comprehensively evaluate the series of outcome indicators, used the RE model to analyze the impact of PP on this indicator, and we hope to explain the phenomenon of excessive heterogeneity. A total of 2762 people were included in the experimental group and 2761 people were included in the control group, of which 145 were positive for pericardial effusion in the experimental group and 642 were positive in the control group. Patients in the experimental group (PP group) were less likely to have pericardial effusion than those in the control group (RR=0.34, 95% CI=0.21-0.55; *P*<0.00001). There was a significant difference between the PP group and the control group in early pericardial effusion (RR=0.15, 95% CI=0.04-1.54; *P*=0.004). In assessing late pericardial effusion and pericardial tamponade, we found that the heterogeneity was very low (I^2^=0%). There were significant differences in the incidence of late pericardial effusion and pericardial tamponade between the PP group and the control group after cardiac surgery (late pericardial effusion: RR=0.06, 95% CI=0.02-0.22; *P*<0.0001; pericardial tamponade: RR=0.17, 95% CI=0.06-0.46; *P*=0.0005). After the abovementioned outcome indicators were combined and analyzed, we found that, compared with the control group, the incidence of pericardial effusion in the whole period and the incidence of pericardial tamponade were significantly different (RR=0.26, 95% CI=0.17-0.39; *P*<0.00001) (Figure 4). Obviously, the PP group was superior to the control group in this index.

### Secondary Outcomes

There was no significant difference in postoperative pulmonary complications between the PP group and the control group (RR=0.99, 95% CI=0.71-1.38; *P*=0.96). The positive muscle strength support demand in the PP group was significantly lower than that in the control group (RR=0.66, 95% CI=0.52-0.85; *P*=0.001). In terms of mortality, length of stay, and ICU time, there was no significant difference between the PP group and the control group (in-hospital time: standard deviation=0.02, 95% CI=-0.18-0.23; *P*=0.83; mortality: RR=0.72, 95% CI=0.32-1.60; *P*=0.42; ICU time: standard deviation=0.34, 95% CI=-0.04-0.67; *P*=0.08). In terms of coagulation function, that of the control group was better than that of the PP group (RR=2.63, 95% CI=1.73-3.99; *P*<0.00001).

### Sensitivity Analysis

**Fig. 5:**
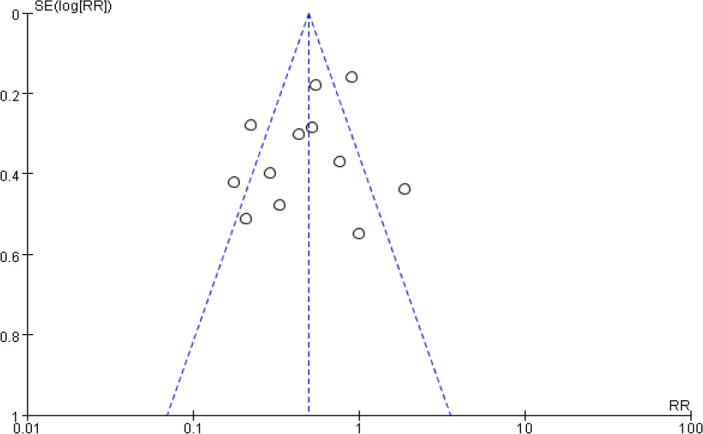
Funnel plot to assess potential bias in the postoperative atrial fibrillation part analysis. RR=risk ratio; SE=standard error.

Because of the high heterogeneity in the analysis of the main outcome indicators, we omitted four RCTs^[17,21,22,24]^ and found that heterogeneity decreased from high to low (I^2^ decreased from 76% to 22%). However, after excluding these trials, the conclusions of the analysis did not change significantly (RR=0.47, 95% CI=0.38-0.59; *P*<0.00001). Funnel plot was used to evaluate the potential bias in POAF analysis (Figure 5). A more uniform distribution on both sides represents a lower risk of potential bias. In addition, we conducted a sensitivity analysis of the patients’ baseline data and surgical variables. The results showed that I^2^ decreased from 76% to 69%, which was similar to the previous heterogeneity and would not affect the conclusion. Therefore, we do not think it is necessary to exclude the studies that cause high heterogeneity.

## DISCUSSION

POAF is reported to complicate 20-40% of cardiac surgeries and 10-20% of noncardiac thoracic surgeries. Once POAF occurs, its complications can be severe or even fatal^[27,28]^. There is no doubt that pericardial effusion is harmful. It can reduce cardiac output, reduce the intensity of ventricular wall motion, and even lead to pericardial tamponade, resulting in unplanned secondary surgery or postoperative cardiac arrest, aggravating the suffering of patients, and increasing medical costs^[29-32]^. In our meta-analysis, the use of PP to control POAF was successfully demonstrated, and this small trauma non-drug approach is worth promoting. Another focus of this meta-analysis was to study the effect of PP on pericardial effusion. We divided pericardial effusion into early pericardial effusion, late pericardial effusion, and pericardial tamponade. Regardless of the type of pericardial effusion, the PP group showed unparalleled advantages (RR=0.26, 95% CI=0.17-0.39; *P*<0.00001).

According to our meta-analysis, PP also has some drawbacks. The coagulation function of the PP group was significantly worse than that of the control group. This may be due to the activation of prothrombin factor after pericardial incision, resulting in stronger coagulation function. This mechanism has not been fully explored. However, this increased coagulation function is a potentially fatal complication for patients after cardiac surgery. At the same time, some studies also reported that after PP, patients had complications such as pericarditis, pleurisy, and even right heart failure^[14,26]^. This makes the patient’s hospital stay longer and medical expenses increase. Therefore, cardiac surgeons need to fully assess the patient’s basic conditions before choosing whether PP is needed. After PP, patients should be closely observed whether there is pericardial effusion or pleural effusion, and the team should be alert to the occurrence of constrictive pericarditis or pleurisy to improve the survival rate and quality of life of patients.

Compared with previous meta-analyses, we obtained more accurate and reliable results on the impact of pericardial effusion^[33-35]^. At the same time, this study has other advantages. Our statistical analysis is based on the effect of RR, and all analyses adopt the RE model. In addition, we were not solely based on CABG for analysis; we included all of the cardiac surgeries using PP RCT. Moreover, this study included a newly released high-quality RCT that provided additional data^[14]^. We believe that this new RCT greatly consolidates our view and is of higher quality and credibility than previous meta-analyses.

Also, because we included some studies where CABG was not performed, the results of this study were similar to those of a previous meta-analysis^[33-35]^, which may mean that reduced incidence of POAF and pericardial effusion after PP may be generally applicable to cardiac surgery.

At present, the application of β-receptor blockers to prevent POAF has become very extensive. β-receptor blockers slow heart rate, weaken myocardial contractility, decrease cardiac output, and slightly lower blood pressure by blocking the cardiac β1 receptor, which can delay the conduction of the sinoatrial node and atrioventricular node, inhibit the self-regulation of myocardial cells, and eliminate supraventricular and ventricular tachyarrhythmias caused by increased self-regulation and reentrant excitation. It can be manifested as the prolongation of the P-R interval of electrocardiogram due to the prolongation of atrioventricular node conduction time^[36,37]^. However, β-receptor blockers may lead to airway pressure increase, hypoglycemia, and other adverse reactions, which may be fatal for postoperative severe patients^[36]^. Therefore, using PP to prevent POAF becomes particularly important.

This study also analyzed some important indicators of routine testing in ICU patients. We found some interesting results. The PP group had no obvious advantages in terms of postoperative pulmonary complications, length of hospital stay, mortality, or ICU time. However, PP shows a great advantage in positive muscle strength support. This may be due to reduced atrial and ventricular pressure after pericardial incision, followed by easier heartbeat and reduced vasoactive drugs. However, the abovementioned speculation is based on this study, which has not been confirmed by other animal or human experiments. Massive hemorrhage is a recognized problem in cardiac surgery^[38]^. There are many causes of hemorrhage during cardiac surgery, including surgical problems or perioperative coagulation disorders^[39,40]^. Once coagulation disorders occur, patients will face major problems such as allogeneic blood transfusion, pericardial tamponade, and even emergency thoracotomy^[38]^. Therefore, it is very important to maintain the stability of perioperative coagulation function. In terms of coagulation function, we found the opposite results. The coagulation function of the control group was significantly better than that of the PP group. This may be due to the activation of the coagulation system and the increase in coagulation factors and thrombin after pericardial incision^[41,42]^. It is necessary to pay special attention to this point in postoperative treatment and adjust the dosage of coagulation drugs according to international ratio in time.

However, this article also has some shortcomings. The overall sample size is relatively small, and the included RCTs did not have drugs to control heart rate before surgery, which may lead to greater bias in POAF analysis. The overall heterogeneity of research was relatively high, but we have reasonably explained this in the sensitivity analysis.

### Limitations

The sample size we included was limited, the number of reports on complications was small, and the use of preoperative drugs was not controlled. More RCT experiments may be needed to answer these questions.

## CONCLUSION

In this systematic review and meta-analysis, PP has shown good results in the prevention of POAF and pericardial effusion and fewer complications, indicating that PP is a safe and effective surgical method, but we still need to pay attention to the potential risk of PP leading to coagulation dysfunction.
